# Germicidal Powers of Vaginal Mucus

**Published:** 1907-07

**Authors:** 


### GERMICIDAL POWERS OF VAGINAL MUCUS.

Clifton Edgar expresses the opinion that the teaching of Bunner and Kronig, which held that the vaginal mucus possessed a bactericidal action, has done more harm than good, because it has been widely misunderstood and generally applied to gonococcus infection. The vaginal mucus has never been proven to possess such an antagonism to the gonococcus. If we accept the frequency of gonorrheal infection in the male as laid down by Blaschko, Ricord and Erb, as only approximately correct, then it must follow that the same infection in the female is more frequent than is generally accepted. It certainly in the author's experience, is commonly met with in private practice. And if this be true, and moreover if it be true that gonorrhea in pregnancy is often overlooked by reason of the absence of uterine and parauterine, and periuterine conditions, then the tendency to consider the pregnant vagina sterile in most instances is dangerous and liable to increase the morbidity of the puerperium, and the cases of ophthalmia neonatorum.—Med. Record.

